# Navigating the Expanded Access Pathway to Investigational Drugs as an Academic Oncologist

**DOI:** 10.1001/jamanetworkopen.2023.0060

**Published:** 2023-02-17

**Authors:** Holly Fernandez Lynch, Tasnim Salam, Patrick Gould, Alison Bateman-House, Laura Kimberly

**Affiliations:** 1 Perelman School of Medicine, University of Pennsylvania, Philadelphia; 2 NYU Grossman School of Medicine, New York, New York

## Abstract

This qualitative study explores academic oncologists’ needs and satisfaction with expanded patient access to investigational drugs.

## Introduction

Expanded access (EA) allows patients to be treated with unapproved medicines outside trials if their physician, the drug company, the US Food and Drug Administration (FDA), and an institutional review board (IRB) sign off. Prior studies^1,2,3,4^ indicate physicians have a positive impression of EA but face challenges coordinating the multistep, multistakeholder process. We sought to understand the resources needed to support academic oncologists making EA requests, explore their satisfaction with other EA gatekeepers, and examine their perspectives on the utility of EA data, hypothesizing that their experiences could illuminate core challenges.

## Methods

Study methods are comprehensively described in the first publication from this data set.^5^ The study was exempted from IRB review by the University of Pennsylvania and approved by the IRB at NYU Langone Health. Participants gave oral permission to be interviewed and recorded at the start of each interview. The study follows the Consolidated Criteria for Reporting Qualitative Research (COREQ) reporting guideline.

## Results

We report interview results from 25 oncologists with prior EA experience at large academic medical centers in the Northeast US. Participant characteristics are described in Table 1, with key findings summarized in Table 2. We did not collect data on participant race, ethnicity, or age.

**Table 1.** Demographic Characteristics of Participating Oncologists^a^

Characteristic	No. (%) of oncologists (N = 25)
Observed gender	
Male	10 (40)
Female	15 (60)
Length of clinical experience, y	
0-5	4 (16)
6-10	5 (20)
11-15	2 (8)
16-20	6 (24)
>20	8 (32)
Estimated No. of oncologist discussions with patients and families about single-patient expanded access (total, 6 y before the COVID-19 pandemic)^b^	
1-5	7 (28)
6-15	10 (40)
16-40	4 (16)
41-74	0
≥75	2 (8)
Unclear	2 (8)
No. of single-patient expanded access requests to IRB (January 1, 2014-January 31, 2020)^c^	
1	11 (44)
2	7 (28)
3	4 (16)
4	1 (4)
≥5	2 (8)
Type of oncology practice	
Adult	15 (60)
Pediatric	10 (40)
Institution	
University of Pennsylvania	6 (24)
NYU Langone Health	1 (4)
Children’s Hospital of Philadelphia	8 (32)
Dana-Farber Cancer Institute	10 (40)

Abbreviations: IRB, institutional review board; NYU, New York University.

^a^ Derived from Gould et al.^5^

^b^ All interviewees were asked to estimate the number of patients for whom they had discussed single-patient expanded access with the patient or family in the 6 years before the COVID-19 pandemic, between 2014 and the start of 2020. Few interviewees were able to provide a precise number, instead offering a general range; some may have included both single-patient expanded access and expanded access protocols for multipatient groups or considered a different time range, despite instructions to the contrary. When interviewees offered a percentage of patients, they were asked to instead provide a count. One interviewee provided only a percentage (“Maybe 15-20%”), and 1 indicated “dozens”; these are reflected in the table as “unclear.”

^c^ These numbers were derived from information provided by each interviewee’s IRB.

**Table 2.** Summary of Key Themes

Theme	Summary	Representative quotations
Frequency of EA discussions driven by available options	Whether oncologists consider EA depends on the pipeline of new drugs, available trials, and recent drug approvals.	“And so for a period of time, that was a conversation for a large portion of patients and then the drug became approved, so that it faded over time.” A08 “I think with the advent of immunotherapy and there being more pipelines in the pharmaceutical industry for immunotherapy products…I think that increased [the] number of times that we talked about compassionate use or expanded access…” P09
	Some oncologists perceive an increase in EA discussions based on social media.	“I mean, I think again, just the connectivity of our families. I think we have had some very promising drugs recently…that have gotten a lot of buzz. And kind of in that gap between FDA approval and studies being closed, a lot of patients have heard about these drugs.” P05
EA as a resource-intensive team effort	Oncologists lack formal EA training but report learning as the need arose, often during fellowship.	“I was a second-year fellow…and a young patient came in [need of a protein] available in France only, and it was not available in the US…I found out on my own about how to get a single-patient IND…” A14 “I was a fellow…I spoke to, I remember this so vividly, an oncologist at the time…and she said [the] patient was too ill to qualify for any clinical trials but there’s this new drug… (now a very old drug) that the NCI is developing and it looks active and if you applied for an EAP you would probably be able to get it.” A04
	Oncologists rely on professional colleagues when considering EA requests but lack formal EA peer review processes.	“I think that we often seek buy-in from our colleagues about pursuing something by expanded access, or we update them that this is what we’re doing.” P03 “Our expectation within our section is that if you were going to treat a patient with a compassionate access agent, that that’s something that we would have discussed clinically, and people would have had an opportunity to weigh in on that. But we don’t have a process way to enforce that.” P07
	Oncologists typically have a general understanding of EA requirements but depend on others for specifics. They often describe a division of EA labor with research and regulatory staff, whose involvement is viewed as critical.	“I mean, drug companies always mystify me sometimes with what they do and they’re all different, but generally, I understand what it takes to get it and I have people here to help me too, thankfully. My research staff will do a lot of the legwork generally. I may have to call a medical somebody or other at some company sometimes.” A12 “It’s true for both institutions I was at and my current institution, there is a team of people who manages the interactions and the paperwork. So, that's such that the physician writes the medical need section and outlines what the treatment plan is going to look like. And then there are regulatory and administrative services that interact with the IRB and FDA.” P08
	Participation in EA requests is routinely described as resource intensive, unfunded, and challenging, with variable institutional support.	“They really don't want us to use [EA]…It’s just using resources that are already stressed in our Cancer Center, and really don’t move the sort of, research enterprise forward at all.” A05 “[The] opportunity costs are huge because everybody says to your unit well you can do this, but you’re going to have to do it with the same people that are responsible for managing the rest of your research portfolio. And do you want to degrade the maintenance of that portfolio to do this for a single patient?” A03
General satisfaction with EA gatekeepers	Oncologists describe company interactions as easiest when there is a preexisting relationship or the company has handled prior EA requests.	“So there are circumstances in which the company is, you’re the 50th person to ask for this. And then it’s not bad. You get a form, they send you to the website, you put a little bit of information in.” P02 “The company, it’s again fortunately, I’ve got a lot of relationships with the companies just because of clinical trials that I run, and so I know the people that actually have the power to make the decision to give me the drug.” A10
	Oncologists typically report successful EA requests to companies, largely attributed to selection bias in terms of which drugs they request for which patients and from which companies.	“[T]here are some companies that historically do not participate in expanded access and therefore are not even worth calling. And there are others that you know if you call, you can get it.” P08 “Most of the recent cases…have had a very good biologic rationale and the company says, ‘We agree.’” P05
	Oncologists find blanket denial policies problematic.	“I don’t like when they’re just like, ‘Yeah, no, our standard is that we don’t give out compassionate use.’” A02 “I think the least acceptable reason which I see all the time but [not recently] is companies who are terrified of having anything to do with kids and so they don’t want anything to do with it.” P02
	Oncologists tend to view most specific company reasons for refusing EA requests as reasonable, including concerns about the following: safety, effectiveness, and lack of data; logistical issues; and possible impact on FDA approval decisions.	Safety, effectiveness, and lack of data “[I would consider an acceptable denial to be] for any sort of safety-related risk, if there was a concern in a particular patient that they would get more harm or any major type of adverse event because of a drug and felt strongly for that, especially if they had some data that that was a high likelihood.” P06 “[T]hey may say, ‘Look, our science doesn’t support your request.’ And that’s a very legitimate concern, they know the mechanism, they know the drug, they know the preclinical data often better than us.” A08 Logistical issues “There was one type of therapy where the technology to develop the treatment is extremely precious and time-sensitive, and they only had enough basically to run their own studies and which is totally understandable.” A10 Possible impact on FDA approval decisions “I guess if they’re worried about where their drug is in terms of the development process and whether anything like this might impact their ultimate approval by the FDA, I can see that, and ultimately, if the greater good is to get the drug approved for larger numbers of people, I understand that.” A15
	Oncologists are largely supportive of FDA’s role in EA, indicating it is not a barrier and noting recent improvements.	“I think that they [FDA] are critical to being a neutral party between the pharmaceutical company and the physician and family and can think maybe more objectively than either of those 2 groups.” P08 “I think it’s really important to have some nationalized mechanism to track the applications and how often is the program being accessed, and is there an emerging safety signal…” A08
	Only a few oncologists perceived FDA’s EA role as perfunctory, redundant, or otherwise challenging.	“[FDA’s involvement in EA] is largely a waste of time. It’s more paperwork. Because they really don’t review….They kind of know if the drug company’s going to give it out, it must be pretty good. I’ve never had the FDA really say no to an expanded access. It's more like a pro forma thing…” A12 “I’m not sure [FDA’s involvement is] a totally necessary step given that these are all reviewed by local IRBs. I think that where you could do one or the other, it could be reviewed by the FDA, not the IRB…” A11
	Most oncologists had few complaints about their EA experiences with IRBs, although some suggested IRB involvement may be redundant.	“Oh, I think it’s important. I think it’s almost as important as any other role of the IRB, because it really is single-patient experimentation and we have to make sure that we do that in a thoughtful way.” P10 “They’re there for patient safety and institutional oversight of me. I’m part of the institution. And I think that’s appropriate that they watch me and they vet me and they don’t let me do something crazy outside their mission.” A01
Mixed views on gathering data from EA	Oncologists tend to view safety data from EA as critical.	“Safety data [are] critically important because it’s going to be used in an entirely different kind of setting and who knows what we're going to find.” A02
	However, they split on the value of EA efficacy data.	“I think that that’s really more the world of the clinical trial development where some of the confounders are more controlled for and so I don’t rely so much on the efficacy data from anecdotal reports of things like expanded access programs where it’s really an assembly of anecdotes. So I wouldn’t weigh efficacy data nearly as heavily as safety data in that setting.” A08 “[I]n my case, it actually turned out to open up a whole opportunity for the drug and the company, because it worked, and it led to other single-patient INDs for the same drug. And now, it’s led to a multicenter clinical trial. So, I think that these can be opportunities to learn about drug activity in a particular rare disease that otherwise might not be studied.” A10
	Oncologists worry about unfunded resource burdens associated with collecting EA data.	“I think one of the barriers is that when you set up your expanded access, it is clear that it is not research. And therefore, you can’t use research dollars to complete the tasks. They have to be clinical dollars….There aren’t people and resources to do the data collection the same way you would on a clinical trial.” P08 “I actually think that if companies want to use expanded access data toward their regulatory approval, they should write a protocol. They should compensate the sites….They can’t try to go around the bush by having us do what’s clinical work and the regulatory work in an uncompensated way.” A07

Abbreviations: A, adult oncologist; EA, expanded access; EAP, Expanded Access Program; FDA, US Food and Drug Administration; IND, investigational new drug; IRB, institutional review board; NCI, National Cancer Institute; P, pediatric oncologist.

Most oncologists reported engaging in infrequent EA discussions with patients and families, usually just once or twice a year, although a few did so much more often. They typically rated their knowledge of EA as moderate to strong and relied on a division of labor in which they served as “quarterback,” handling initial communications with companies and provision of clinical information while relying on research and regulatory staff for assistance with process, paperwork, and logistics. Participants noted that EA could divert attention from running trials and research funds could not be used because EA is not considered research, leading to some institutional discouragement.

Oncologists generally did not experience companies or the FDA as barriers and reported that both entities typically answer EA requests within a few days. When describing acceptable reasons for company denial, oncologists listed safety or effectiveness concerns, lacking an appropriate formulation or manufacturing capacity, and fear of adverse events negatively influencing marketing approval. Oncologists disagreed with blanket policies denying EA, viewing that approach as insufficiently nuanced. Most supported the FDA’s involvement in EA because of the perceived need for independent review to mitigate physician and company biases, avoid the “wild west” in inappropriate use of unapproved drugs, require safety tracking, and avoid trial interference. Oncologists did not view the requirement for IRB authorization of EA as burdensome, but some perceived IRBs as adding nothing beyond FDA’s review, whereas others noted a unique IRB role to ensure consent or provide institutional oversight.

Oncologists viewed EA safety data as critical but diverged on the value of EA efficacy data. Several noted that efficacy data would be less useful because they are not generated through a well-designed trial; others indicated that efficacy data could identify unexpected results, inform future research, or provide insight for rare diseases facing trial challenges because of small populations. Many oncologists expressed concern about collecting any EA data due to added burden, with a few reporting unrealistic company contract requirements in this regard that often went ignored.

## Discussion

Our findings are limited to the perspectives of oncologists practicing at a few large academic medical centers; those practicing in other settings may have different views, especially dependent on their access to supportive resources and company contacts. The fact that oncologists in our sample view EA requirements as burdensome but nonetheless reasonable seems to acknowledge that this pathway is inevitably more resource intensive than routine clinical care, perhaps leading to infrequent use. Given competing resource demands and uncertain patient benefit, it is unclear whether resources should be devoted to further facilitating EA vs simply eliminating unnecessary burden. Improving the possibility of learning from EA through data collection—an area currently gaining attention—could strengthen the value proposition but blurs the line between EA and research.^6^ Future analyses should prioritize developing a better understanding of EA’s costs and benefits, including in different diseases and practice settings, to inform resource allocation and policy decisions.

## Notes

Supplement.
Data Sharing Statement
