# Supplementary material for: Navigating the Expanded Access Pathway to Investigational Drugs as an Academic Oncologist
Source: JAMA Netw Open. 2023 Feb 17;6(2):e230060. doi: 10.1001/jamanetworkopen.2023.0060 (PMC9938427; doi:10.1001/jamanetworkopen.2023.0060)
Supplement: Supplement. — Data Sharing Statement [file jamanetwopen-e230060-s001.pdf]

Data Sharing Statement

Fernandez Lynch. Navigating the Expanded Access Pathway to Investigational Drugs as an Academic Oncologist. *JAMA Netw Open*. Published February 17, 2023.
doi:10.1001/jamanetworkopen.2023.0060

Data
Data available: No

Additional Information
Explanation for why data not available: Interview participants did not consent to data sharing. Requests for deidentified transcripts may be submitted to the corresponding author for consideration in collaboration with the IRBs at the University of Pennsylvania and NYU Langone Health.
